# Electrodeposition of ternary compounds for novel PV application and optimisation of electrodeposited CdMnTe thin-films

**DOI:** 10.1038/s41598-020-78066-y

**Published:** 2020-12-08

**Authors:** A. E. Alam, O. I. Olusola, D. A. L. Loch, K. Shukla, W. M. Cranton, I. M. Dharmadasa

**Affiliations:** 1 Materials and Engineering Research Institute, Sheffield Hallam University, Sheffield, S1 1WB UK; 2 Department of Physics, School of Science, Federal University of Technology, Akure (FUTA), Ondo State, P. M. B. 704, Akure, Nigeria

**Keywords:** Electrical and electronic engineering, Solar cells, Electronic devices, Solar cells, Electrochemistry

## Abstract

Growth of polycrystalline CdMnTe ternary compound thin films has been carried out using cathodic electrodeposition technique at different cathodic potentials. The range of the cathodic potentials used in this work has been chosen according to the cyclic voltammogram results. The CdMnTe thin films were electroplated from electrolyte containing CdSO_4_, TeO_2_ and MnSO_4_ in an acidic aqueous medium. Glass/fluorine-doped tin oxide (FTO) substrates have been used to electrodeposit the semiconductor layers. The structural, compositional, morphological, optical and electrical properties of the CdMnTe thin films were studied using X-ray diffraction (XRD), Sputtered neutral-mass spectroscopy (SNMS), Scanning electron microscopy (SEM), UV–Vis spectroscopy and Photo-electro-chemical (PEC) cell measurements respectively. The primarily grown as-deposited (AD) layers went through two different post-growth surface treatment conditions- heat-treated in air in the presence of CdCl_2_ (CCT) and heat-treated in air in the presence of GaCl_3_ (GCT). Results from the XRD indicated the polycrystalline nature of the electrodeposited films. The electroplated films have cubic crystal structures and the preferred orientation was found to be along the (111) plane of CdMnTe. Inclusion of Mn has been qualitatively observed using SNMS measurement. The optical energy bandgaps of the thin films were found to be varying between ~ 1.90 and ~ 2.20 eV. Though all the layers after post-treatment showed p-type electrical conduction, both p and n-type conductivity were obtained at different cathodic potentials for as-deposited materials. Comparison of the deposited layers to other electrodeposited ternary compounds has also been discussed.

## Introduction

CdTe based thin-film photovoltaic (PV) cells are currently leading the thin-film PV market. Though, First Solar-USA is in the commercial forefront with 18.7% module efficiency at present, the first ever commercial manufacturing of CdTe-based solar cells was successfully executed by British Petroleum (BP) in 2001 with their 10.6% efficient ~ 1 m^2^ solar modules^1,2^. Since First Solar-USA uses a modified Close Space Sublimation (CSS) technique for the manufacturing of their solar cells, CSS has become the leading fabrication method worldwide and a vast research emphasis has been given on cells based on this technique^3^. Though, apart from CSS, other growth techniques used for CdTe-based solar cell fabrication such as Chemical vapour deposition (CVD), Sputtering, Molecular beam epitaxy (MBE) etc. have also been experimentally explored, the only other technique that could be commercially implemented for large scale CdTe-based solar cells was BP's Electrochemical deposition (ED)^2,4–6^. The untimely exit of BP from their CdTe-based solar project around 2002 and later their complete termination of the solar venture in 2011 slowed down the flourishment of this excellent technique, yet this is still one of the only two commercially successful fabrication techniques and requires very economical manufacturing set-up compared to the others^7^. As a matter of fact, considering the simplicity of the set-up, deposition process continuity, doping simplicity, self-purification capability, scalability, manufacturability, economic viability, likelihood of Cd-containing waste reduction and necessity of comparatively less number of production lines, electrodeposition is the most suitable fabrication technique that can be feasibly executed in the developing world^8–13^.

**Figure 1:**
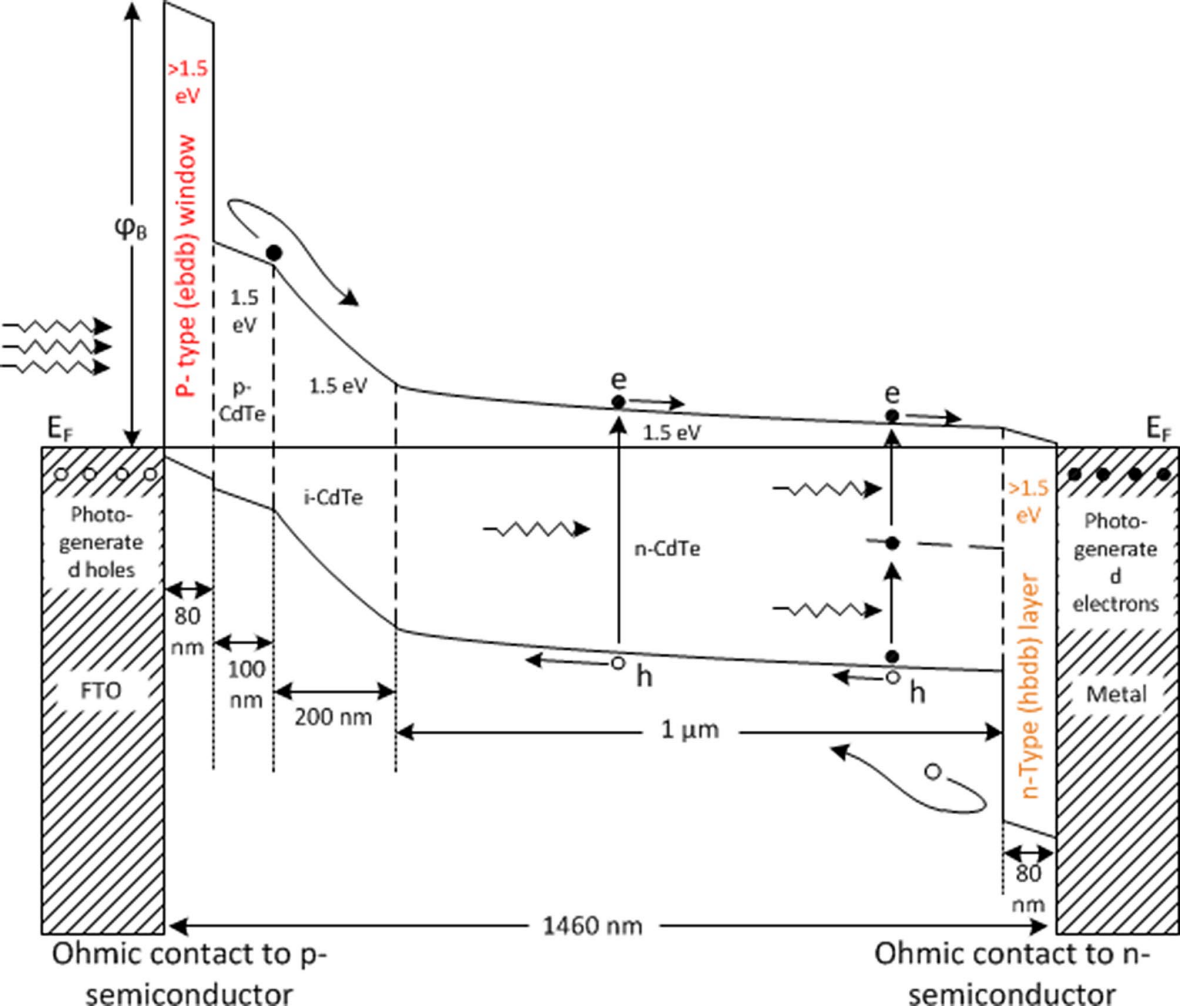
Energy band diagram drawn to scale for a short-circuited multi-layer solar cell based on p-type window material (p^+^-p-i-n–n^+^ structure). P^+^ and n^+^ layers act as electron back diffusion barrier (ebdb) layer (or Hole Transport Layer- HTL), and hole back diffusion barrier (hbdb) layer (or Electron Transport Layer- ETL) respectively.

Currently, Solar energy research group, at Sheffield Hallam University holds the record of highest cell efficiency for CdTe-based electrodeposited PV, where graded bandgap structure has been implemented to fabricate the devices^14^. Graded bandgap (GBG) multi-layered solar cells are highly promising in designing the next generation solar devices. It has been conceptualised by our group that, GBG devices can be constructed based on two approaches in terms of the electronic conduction type of the window material; i) fabricated on n-type window and ii) fabricated on p-type window. Though our current highest efficiency for CdTe-based GBG solar cells are achieved from n-window devices, cells fabricated on p-type windows have a potential to achieve higher performance due to the possibility of producing higher open circuit voltage (V_oc_) resulted from a higher potential barrier^15,16^. This has been experimentally demonstrated by achieving Voc ≈ 1.17 V with GaAs/AlGaAs and Voc ≈ 1.00 V with Perovskite based solar cells^17,18^. Therefore, the group now aims to achieve this device structure for CdTe-based low-cost ED solar cells. A prospective device design as per Fig. 1 has been presented in our previous publication^19^.

In order to implement this conceptual solar device, the primary task is to search for a suitable p-type semiconductor material that can be electrodeposited having a bandgap higher than that of the absorber material, which in this case is > 1.45 eV of CdTe. Moreover, for the best outcome of the device it is also vital that the material has a reasonable crystallinity along with low or no intermediate defect levels present within the bandgap range for better career transport and accurate Fermi level positioning respectively.

In search of a suitable p-type window layer, previously Magnesium (Mg) incorporated CdTe (CdTe:Mg), a ternary compound has been grown by ED which demonstrated a p-type electrical conductivity along with a bandgap of ~ 2.80 eV^20^. The reason to choose Mg to incorporate to the CdTe layer was mainly that Magnesium Telluride (MgTe) has shown a very small (0.7%) lattice mismatch with CdTe and demonstrated a higher bandgap^21,22^. However, the layers exhibited a dramatic collapse of crystallinity with the incorporation of Mg and have been seen to give rise to an intermediate defect level at ~ 1.45 eV which may potentially pin the Fermi level and lower the barrier height. Hence, the layer lacked all the required properties to be used as the p-window layer of the designed device.

This work focuses on incorporating Manganese (Mn) in the CdTe in order to form an electrodeposited ternary compound CdMnTe (CMT) to be used as the p-type window layer for CdTe-based PV cells. Once again, Mn has been chosen to incorporate due to the high lattice matching of MnTe with CdTe^23^. Moreover, CdMnTe is increasingly being used as a suitable material for different electronic applications^24,25^. This project is however the first effort to electrochemically deposit CdMnTe layers aiming for CdTe-based solar devices.

## Experimental details

Using GillAC ACM potentiostat in potentiostatic configuration, CdMnTe ternary compound semiconductor was cathodically electrodeposited on glass/FTO conducting glass substrates. The conducting substrate used in this work is TEC 7 with a sheet resistance of 7 Ω/square. One vital factor which is essential in growing a uniform semiconductor material with proper adherence to the substrate is that the substrate surface must be thoroughly cleaned. To accomplish this, they were washed with soap solutions using cotton buds followed by a further rinsing action using de-ionised water. The surfaces were finally dried under a nitrogen gas flow, before being applied as the working electrode in the electrodeposition (ED) set-up.

The CdMnTe thin films were deposited from electrolyte containing 1.00 M CdSO_4_ (99.999% purity), 5 ml of dissolved TeO_2_ (99.995% purity) and 0.12 M of MnSO_4_ (99.996% purity) solution in 400 ml of de-ionised water. The dissolved TeO_2_ solution was prepared by adding 2 g of TeO_2_ powder to 200 ml of de-ionised water. Since TeO_2_ cannot dissolve completely in water, 30 ml of concentrated H_2_SO_4_ was added to the TeO_2_ solution to aid its solubility. The prepared TeO_2_ solution was subjected to continuous stirring and heating for ~ 45 min so as to obtain a very clear TeO_2_ solution devoid of powder^26^. After preparing 1.00 M of CdSO_4_ in 400 ml of de-ionised water, it was continuously stirred to ensure dissolution of the chemical. After the dissolution of CdSO_4_, 5 ml of TeO_2_ and 0.12 M of MnSO_4_ was finally added to the solution to prepare the electrolytic bath. The pH value of the deposition electrolyte was maintained at 2.00 ± 0.02 by using either NH_4_OH or H_2_SO_4_. The growth temperature of the electrolytic bath was ~ 85 °C and the solution was moderately stirred using a magnetic stirrer.

Before the commencement of CdMnTe layer growth, cyclic voltammetry study was carried out to determine the approximate deposition potential for CdMnTe thin films. The ED- CdMnTe thin films were characterised for their structural properties using X-ray diffraction (XRD) technique. In order to confirm Mn inclusion to the deposited layers, sputtered neutral mass spectrometry (SNMS) measurement has been carried out on a HIDEN Analytical SIMS Workstation utilising a quadrupole mass spectrometer. The electrical conductivity type of the ED-CdMnTe thin films was determined by using photo-electro-chemical (PEC) cell measurements. Scanning electron microscopy (SEM) was used in studying the surface morphology of the electrodeposited CdMnTe thin films. The SEM measurements were carried out by using Quanta 3D FEG NanoSEM equipment. The optical properties of the CdMnTe films were studied using Carry 50 Scan UV–Visible spectrophotometer. In order to confirm the p-type electronic conduction type of the CdCl_2_ treated CdMnTe layers, both ohmic and rectifying behaviours were explored using Au and Al electrical contacts respectively. Required structures were fabricated using glass/FTO/p-CdMnTe/Au and glass/FTO/p-CdMnTe/Al and measured by using a computerised 619 Electrometer/Multimeter current–voltage (I-V) measurement system (Keithley Instruments Inc., OH, USA).

## Summary of results

### Cyclic voltammetry

Cyclic voltammetry studies were performed in an aqueous solution that contains 1.00 M CdSO_4_, 0.12 M MnSO_4_ and 5.0 ml of dissolved TeO_2_ solution at a pH of 2.00 $\pm$ 0.02 in 400 ml of de-ionised water. An FTO coated glass substrate was used as the working electrode to study the mechanism of deposition of CdMnTe thin films. A computerised GillAC potentiostat was used to carry out this voltammetric study at a sweep rate of 180 mV min^−1^. In this technique, a range of cathodic potentials from 0 to 2000 mV was applied across the electrolyte through the electrodes in a two-electrode system. In other words, the FTO working electrode is negative with respect to the carbon electrode. The potentiostat was used in monitoring the current through the electrolyte as the magnitude of the voltages between electrodes are varied. In the forward cycle, positive ions in the electrolyte receives electrons from the cathode at appropriate voltages to neutralise and deposit on the cathode. During the reverse cycle, at appropriate voltages the deposited layer on the cathode dissolves into the electrolyte donating electrons to the cathode and moving positive ions into the electrolyte. Therefore, the electric current during the reverse cycle is opposite to that of the forward cycle.

**Figure 2:**
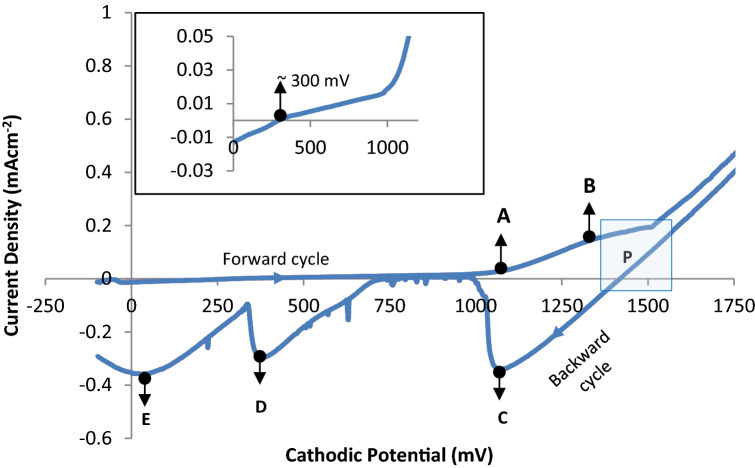
Cyclic voltammogram of electrolyte containing 1.00 M CdSO_4_, 0.12 M MnSO_4_ and 5.0 ml of dissolved TeO_2_ solution (pH = 2.00 ± 0.02, T ~ 85 °C). (Inset shows the transition voltage at which Te begins to deposit).

The redox potential (E_o_) of Te, Cd and Mn are ~  + 0.59, -0.40 and -1.19 V respectively (with reference to standard H_2_ electrode). Since Te shows a more positive redox potential than Cd and Mn, it is therefore expected to deposit first. A typical cyclic voltammogram for FTO-coated glass substrate as cathode in the prepared electrolyte is shown in Fig. 2. The forward curve illustrated at the inset of Fig. 2 shows that Tellurium (Te) begins to deposit at $\sim$ 300 mV. It has been shown that Te being a more noble element deposits first according to Eq. (1). Cathodic deposition of Te at this voltage range is consistent with our previous works where same precursor for Te has been used at similar concentration^26^.

1 $${\text{HTeO}}_{{2}}^{ + } + {\text{4e}}^{ - } + {\text{3H}}^{ + } = {\text{Te}} + {\text{2H}}_{{2}} {\text{O}}$$

As shown in Fig. 2, a rise was observed in the forward current density at ~ 1100 mV (point A), this signifies the initial deposition of Cd on the cathode according to the chemical reaction shown in Eq. (2). At this point the deposition of binary CdTe initiates to take place which is consistent to our previous works on CdTe electrodeposition with sulphate precursor^27^.

2 $${\text{Cd}}^{{{2} + }} + {\text{2e}}^{ - } = {\text{Cd}}$$

Since the E_o_ of Cd is more positive than Mn, Cd starts to deposit after Te and before the deposition of Mn. The rise in current density at ~ 1100 mV reaches its first peak around point B which is at ~ 1300 mV. At this point, there is the deposition of Mn on the cathode according to Eq. (3).

3 $${\text{Mn}}^{{{2} + }} + {\text{2e}}^{ - } = {\text{Mn}}$$

At a cathodic potential above ~ 1300 mV, there seems to be stability in the forward current density from ~ 1320 mV to 1450 mV. Within this voltage range, co-deposition of Te, Cd and Mn takes place and hence the CdMnTe semiconductor compound starts to deposit on the cathode according to the chemical reaction shown in Eq. (4), which is a summation of Eq. (1), (2) and (3).

4 $${\text{HTeO}}_{{2}}^{ + } + {\text{3H}}^{ + } + {\text{Cd}}^{{{2} + }} + {\text{Mn}}^{{{2} + }} + {\text{8e}}^{ - } = {\text{CdMnTe}} + {\text{2H}}_{{2}} {\text{O}}$$

The rectangular box labelled ‘**P**’ represents the selected voltage range (between ~ 1300 to ~ 1560 mV) to grow CdMnTe layers according to this experimental result. Cd-rich CdMnTe materials should grow near the end of this potential range. From the reverse cycle of the I-V curve shown in Fig. 2, the point of transition from the positive current density axis to the negative is ~ 1450 mV and the negative current reaches its first broad peak at point C. This broad peak indicates the dissolution of both elemental and reacted Mn and Cd from the layer formed on the cathode. The dissolution of Te from the surface of the cathode occurs at the broad peaks labelled D and E. Two broad peaks must be representing two phases of Te, presumably oxidised Te and elemental Te respectively. Thus, cyclic voltammetry technique is a vital tool which helps to determine the approximate growth voltage (V_g_) range to deposit CdMnTe thin films. The deposition mechanism of the thin-film layers depending on the varied cathodic voltage and the resultant changes in the layer properties are further explained in “PEC cell measurement for electrical conductivity type” section.

### Structural study using X-ray diffraction (XRD) technique

**Figure 3:**
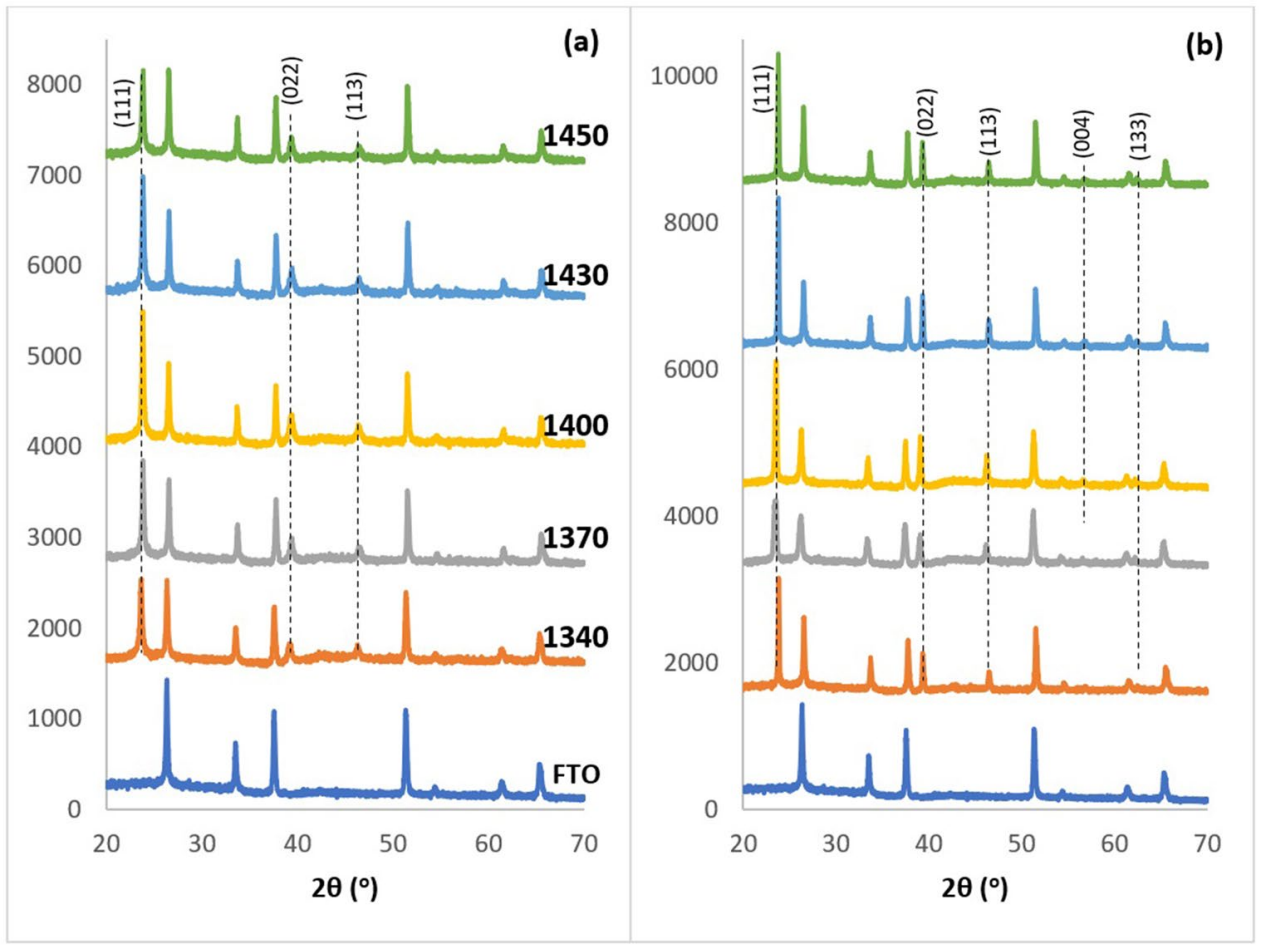
XRD patterns of **(a)** as-deposited and **(b)** CdCl_2_ treated CdMnTe thin film layers grown at V_g_ range from 1340 to 1450 mV.

Structural analysis of the as-deposited (AD) and CdCl_2_-treated (CCT) CdMnTe layers at different growth voltages were carried out using Philips PW X’Pert Pro diffractometer with a Cu-Kα monochromator having a wavelength of 1.54 Å, where the source tension and current have been kept as 40 kV and 40 mA respectively. XRD spectroscopy has been carried out on AD and CCT samples grown at 1340, 1370, 1400, 1430 and 1450 mV cathodic potentials. Figure 3 shows the XRD spectra for both AD and CCT CdMnTe layers grown at different growth voltages. It also shows the XRD pattern of the FTO substrate to aid identification of CdMnTe peaks arising from the layers. Three clear peaks (111), (022) and (113) can be observed for AD-CdMnTe layers showing its polycrystalline nature. In this work, XRD peak identification has been carried out indexing with the ICDD reference file 98–017-4576 for cubic phase.

After the heat treatment with CdCl_2_ at 400 °C for 20 min in air, peak intensities have increased and two more peaks, (004) and (133) emerged indicating improvement of crystallinity. It is clear that the highest crystallinity is observed when CdMnTe layers are grown at 1430 mV. This voltage (1430 mV) has been taken as the optimised growth voltage for CdMnTe under the experimental conditions used in this work, and the other characterisations were carried out only on the materials grown at that voltage. Here, it is to be noted that, CdMnTe is a ternary compound having a very similar crystal structure to CdTe; hence the differentiation of the XRD spectra can be impossible at times. Since, the electrodeposition bath has an excess of Cd precursor, and Mn is the most electro-negative element to deposit, CdTe can well be formed alongside CdMnTe. Inclusion of Mn can therefore be considered as a dopant replacing Cd sites, and the deposited layer could consist of a mixture of CdTe and CdMnTe phases. Due to the similarities in their XRD spectra, there can be overlaps of peak intensities too. Compositional and optical studies carried out in later sections have discussed this issue for further clarification.

**Figure 4:**
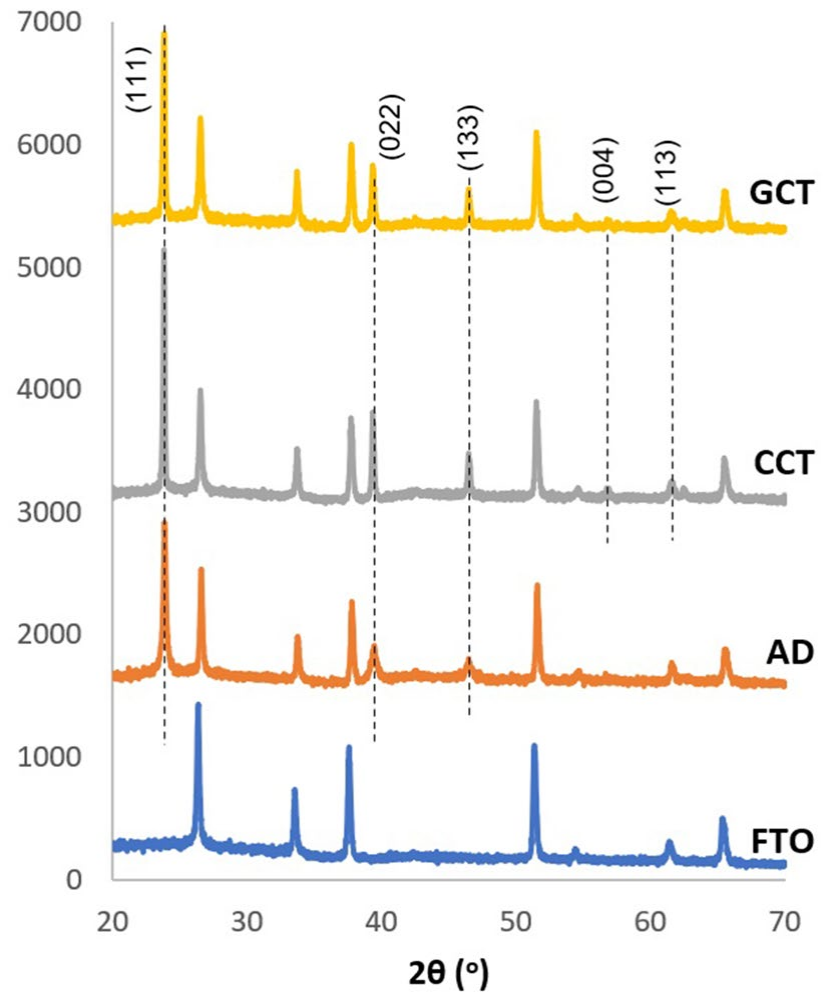
XRD spectra of as-deposited (AD), CdCl_2_-heat treated (CCT) and GaCl_3_ heat-treated (GCT) CdMnTe layers grown at a cathodic potential of 1430 mV.

The layers grown at optimum growth voltage of 1430 mV were tested for two different post-growth treatments, namely CdCl_2_ treatment (CCT) and GaCl_3_ treatment (GCT) based on the notion of previous boost of solar-cell performance observed in literature due to these treatments^26^. In both the cases, the treated layers were heated in air for 20 min at 400 °C. As shown in Fig. 4, the best crystallinity is found for layers treated under CCT conditions.

### Compositional study with sputtered neutral mass spectroscopy (SNMS)

In order to determine the inclusion of Mn in the electroplated layers, standard XPS measurements were carried out. However, these measurements failed to detect any Mn signal, most probably due to the inadequate sensitivity of the measurement instrument in detecting low level of Mn in the layers.

**Figure 5:**
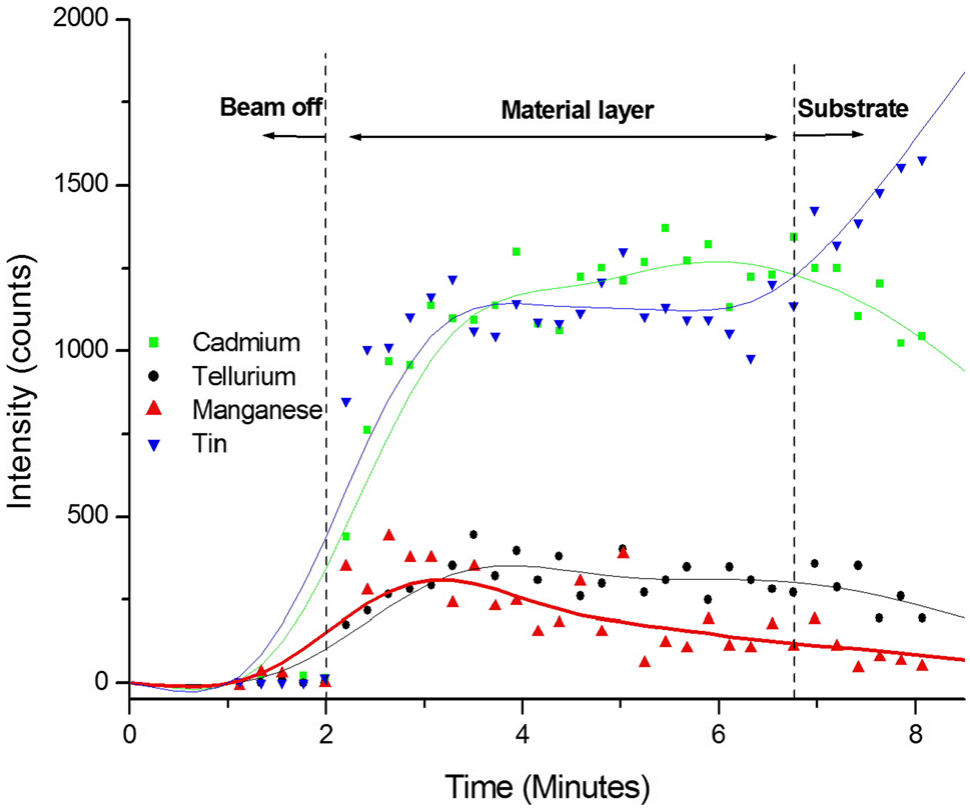
Sputtered neutral mass spectra for the CdCl_2_-treated CdMnTe sample grown at 1430 mV with corresponding 5-points Fast Fourier Transform (FFT) smoothing.

Therefore, to qualitatively demonstrate the inclusion of Mn in the deposited layer, sputtered neutral mass spectroscopy was utilised. Similar to secondary ion mass spectroscopy (SIMS); an Ar ion beam was used to sputter the material layer. An additional ioniser behind the orifice of the mass spectrometer ionises the neutral flux to make it detectable by a mass spectrometer. As the incident angle of the beam is 45° to the sample surface, data was only collected from within a gate area of 100 × 100 μm^2^, to reduce the noise from the sputter trench sidewalls with increasing depth. The total raster area was 800 × 800 μm^2^. The beam parameters were a beam current of 100 µA and an acceleration voltage of 5 keV. In addition to the layer material isotopes (^55^Mn, ^114^Cd, ^130^Te), the tin isotope ^120^Sn was also measured to identify the beginning of the underlying FTO substrate. The sample that has been used for this study is the CCT-CdMnTe layer grown at 1430 mV cathodic potential. In Fig. 5 it can be seen that, in the first minute of the measurement the Ar ion beam remained switched off. The ioniser was in operation to measure the background signal. Once the beam is turned on, all observed isotopes show enhanced signal. After 3 min, the Mn signal reaches its maximum, before slowly reducing to the background level upon reaching the substrate. This indicates that Mn is not evenly distributed throughout the layer but concentrated predominantly to the upper levels. Te exhibits an even distribution over the whole layer, while Cd reaches the maximum intensity close to the substrate surface. The tin signal observed during the sputtering of the layer, can be related to holes in the layer exposing the substrate. However, once the substrate surface is reached, the Sn signal intensifies greatly while the layer material signals drop significantly. Due to the thinness of the layer, the sputter trench thickness could not be measured with enough confidence to report the depth. However, the composition of the layer is qualitatively found to be a Cd-rich CdTe layer with some inclusion of Mn into it. This qualitative composition is pertinent to the cyclic voltametric study carried out in “Cyclic voltammetry” section where co-deposition of Cd, Mn and Te is presumably about to take place in a Cd-rich manner at ~ 1430 mV cathodic voltage.

### Optical absorption study

**Figure 6:**
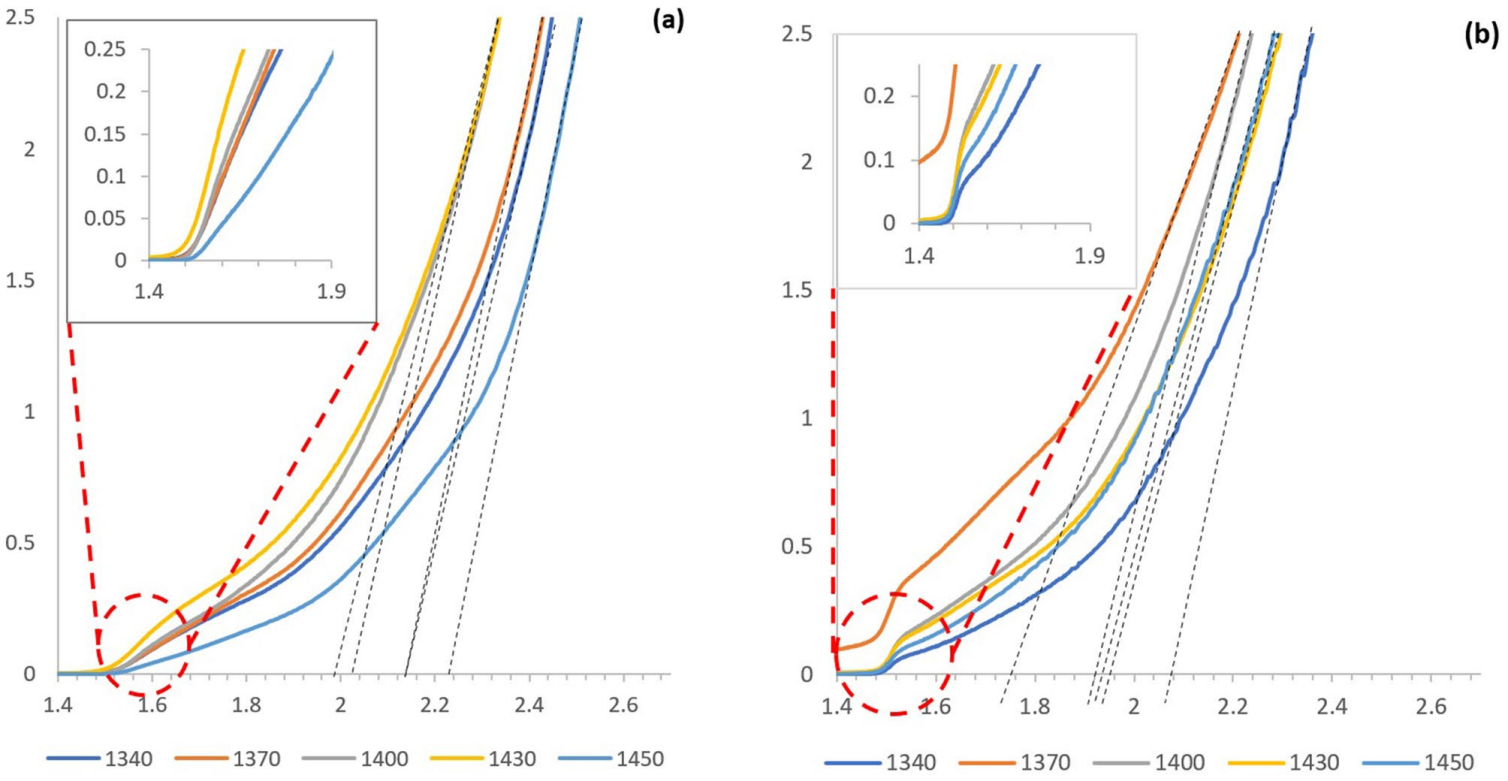
Optical absorption graphs for **(a)** as-deposited (AD) and **(b)** CdCl_2_-treated CdMnTe layers grown within the range of 1340 to 1450 mV. Inset is the sub-bandgap point of absorption at ~ 930 nm.

One key reason of exploring CdMnTe in this work is due to the fact that the energy bandgap of the material is tuneable and wider bandgap than CdTe thin films can be obtained^28^. With the wide bandgap, it can serve the purpose of a p-type CdMnTe layer, to be used as a p-type window material in novel graded bandgap solar cells. Here, the bandgap of the material has been determined using an alternative Tauc plot, where square of absorbance (*a*^*2*^) is plotted against photon energy (*hv*)^26^. The intersection of extrapolated tangent line derived from the curve with the *hv*-axis, gives the direct bandgap (E_g_) of the studied semiconductor material. The absorbance data is collected using a Carry 50 scan UV–Vis spectrophotometer within the wavelength of 300 nm to 1000 nm. Figure 6 shows the *a*^*2*^ vs *hv* plots of CdMnTe layers grown at different cathodic potentials, where bandgap values vary between 1.72 eV and 2.22 eV. However, both the AD and CCT samples illustrate a sub-bandgap point of absorption for the material at ~ 930 nm, which matches the typical absorption wavelength of CdTe (Eg ≈ 1.45 eV). It is also noticeable that post-growth treatments make the mid-bandgap absorption edge sharper. Hence, it can be confirmed that, the XRD spectra found in “Structural study using X-ray diffraction (XRD) technique” section is contributed by both CdTe and CdMnTe crystals.

**Figure 7:**
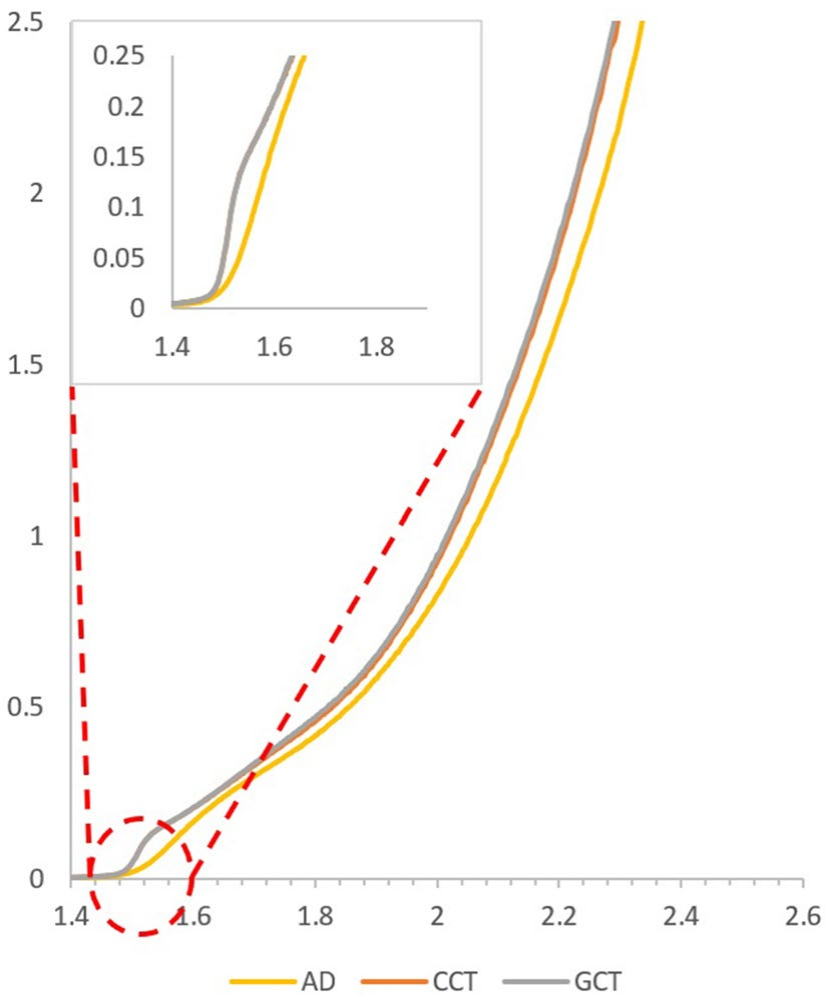
Optical absorption graphs for as-deposited (AD), heat-treated (HT) and CdCl_2_-heat treated CdMnTe layers grown at 1430 mV.

Figure 7 shows the *a*^*2*^ vs *hv* plot for AD, CCT and GCT layers grown at 1430 mV and the bandgap values produced for these layers are all ~ 1.95 eV, whereas samples grown at other voltages have shown quite a significant deviation in bandgaps upon post-growth treatment.

This is further shown in Table 1, where it is evident that an overall decrease in the bandgap is observed after post-growth treatment, though bandgap of the materials grown at 1430 mV stayed almost the same regardless of the treatment. This gives an additional confirmation about the optimum quality of the layers grown at 1430 mV.

**Table 1:** Bandgap of the CdMnTe layers electrodeposited at different growth voltage (Vg) and post-growth treatments.

Vg (mV)	AD-CdMnTe (ev)	CCT-CdMnTe (ev)	GCT-CdMnTe (ev)
1340	2.12	2.05	–
1370	2.12	1.72	–
1400	2.02	1.92	1.92
1430	1.94	1.96	1.96
1450	2.22	1.94	2.20

### Electrical properties of CdMnTe Layers

#### PEC Cell Measurement for electrical conductivity type

**Figure 8:**
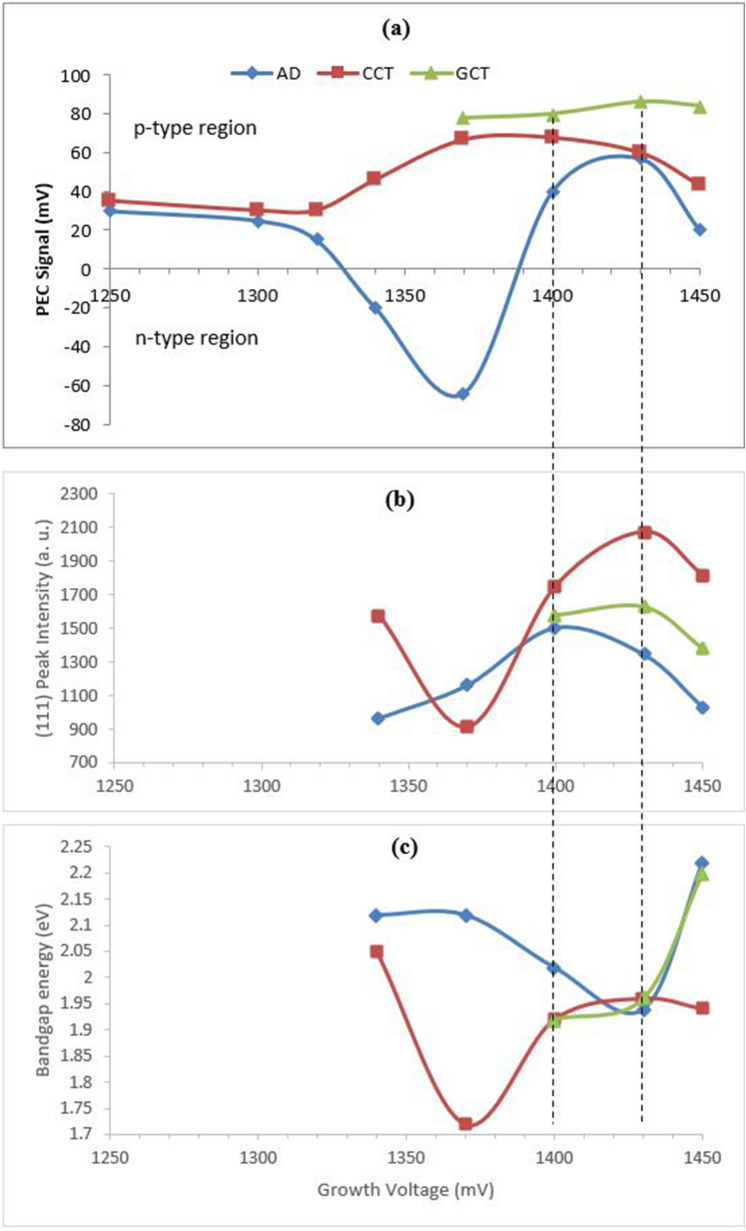
Variation of **(a)** PEC signal, **(b)** XRD intensity of (111) peak and **(c)** bandgap energy as a function of cathodic deposition potential for as-deposited (AD), CdCl_2_-treated (CCT) and GaCl_3_-treated (GCT) CdMnTe layers grown between 1250 – 1450 mV cathodic potentials.

CdTe on its own shows p-type electrical conductivity when grown with Te-richness^16^. Hence, during electrodeposition, CdTe changes its conduction type from p to n, due to the greater incorporation of cadmium at larger cathodic voltages surpassing Te. Here in Fig. 8 (a), the AD layers change their conductivity type from p to n at ~ 1330 mV. All the points are the average of three individual measurements. At ~ 1330 mV the incorporation of Mn is low, consistent to the observation from the cyclic voltammogram in “Cyclic voltammetry” section. The effect of alloying CdTe with Mn on electrical conduction type is visible at ~ 1370 mV of cathodic potential, as the AD layers show an interesting trend of turning p-type from n-type again. This shows the effect of Mn incorporation in turning the conductivity type of the material to p-type. Comparing the change of conductivity type with the (111) peak intensity in Fig. 8 (b) and bandgap values in Fig. 8 (c) it is observed that, the best crystallinity and most stable bandgap value that indicates the quality of the material is found in the cathodic potential range of 1400 mV to 1430 mV. After the CdCl_2_ (CCT) and GaCl_3_ treatment (GCT) all the layers become p-type again and at ~ 1430 mV the treated layers exhibit the highest preferential peak intensity for (111) plane and a very stable bandgap of ~ 1.95 eV. The reason layers grown below ~ 1370 mV before significant Mn inclusion become p-type after CCT can be that, during the chloride treatment, acceptor like defects created at the top of the valence band cause p-type doping on the skin of the polycrystals, and the surface-sensitive nature of PEC picks that up^26,28,29^. For the same reason it is commonly observed in literature that p-type PEC signal is obtained even for Cd-rich electrodeposited CCT-CdTe layers^30–32^. Therefore, to further confirm the conductivity type of the grain interior both ohmic and rectifying behaviours were explored forming Au and Al electrical contacts respectively. However, the acidic nature of GaCl_3_ treatment leaves the layers grown at lower potentials (than 1370 mV) damaged and hence ineligible for characterisation. Since for the application on CdMnTe as the window layer to the proposed GBG solar cells, conduction type of the layers need to be p-type having reasonable optimised crystallinity with bandgap more than 1.50 eV, CCT-CdMnTe grown at ~ 1430 mV can be considered as the suitable layer for the purpose.

#### DC conductivity measurement of CdMnTe layers

**Figure 9:**
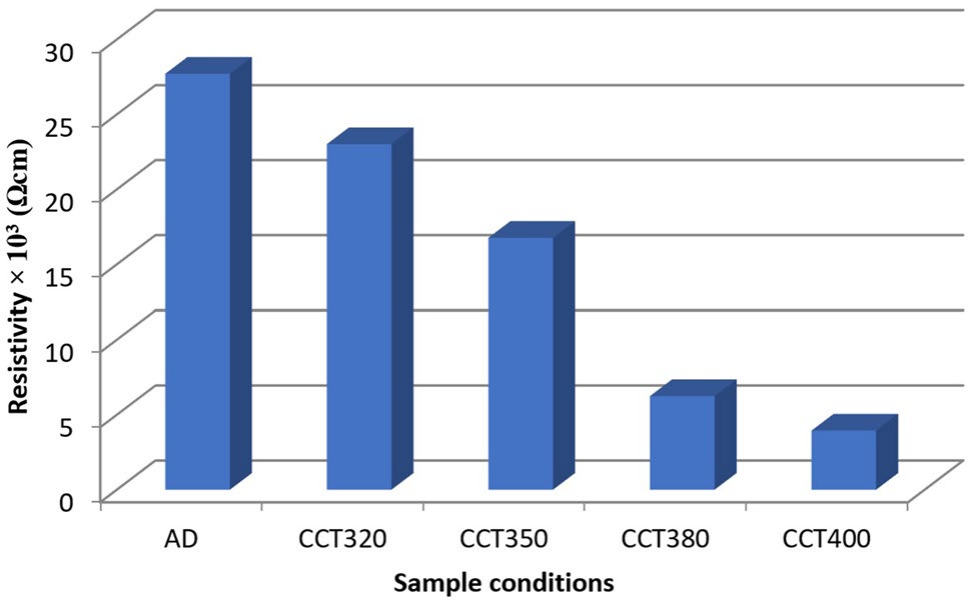
Effect of heat-treatment temperature on the resistance of CCT-CdMnTe layers.

Since the p-type semiconductors form ohmic contacts with Au when Fermi level pinning is not existent, glass/FTO/p-CdMnTe/Au structures were used to explore their electrical behaviour and estimate DC electrical conductivity. 100 nm thick and 0.20 cm diameter circular Au contacts were sputtered on the optimised samples grown at 1430 mV and CdCl_2_ treated at different temperatures. For the measurements, the layers were heat treated at 320, 350, 380 and 400 °C for 20 min in the presence of CdCl_2_. All the contacts showed ohmic behaviour in their I–V characteristics. Moreover, in Table 2 and Fig. 9, effect of temperature in post-growth CdCl_2_ treatment on the resistivity of the optimised layer has been demonstrated, which shows that CdCl_2_-annealed samples treated at 400 °C for 20 min shows lowest resistivity hence highest electrical conductivity.

**Table 2:** Resistivity and conductivity data of the CdMnTe layers CdCl_2_-treated at different temperatures.

Sample	Resistivity, ρ (Ωcm)	Conductivity, σ (Ωcm)^-1^
AD	27.71 × 10^3^	3.61 × 10^–5^
CCT320	23.02 × 10^3^	4.34 × 10^–5^
CCT350	16.77 × 10^3^	5.96 × 10^–5^
CCT380	6.25 × 10^3^	16.01 × 10^–5^
CCT400	3.94 × 10^3^	25.36 × 10^–5^

#### Rectifying behaviour of Schottky diodes fabricated with CdMnTe layers

**Figure 10:**
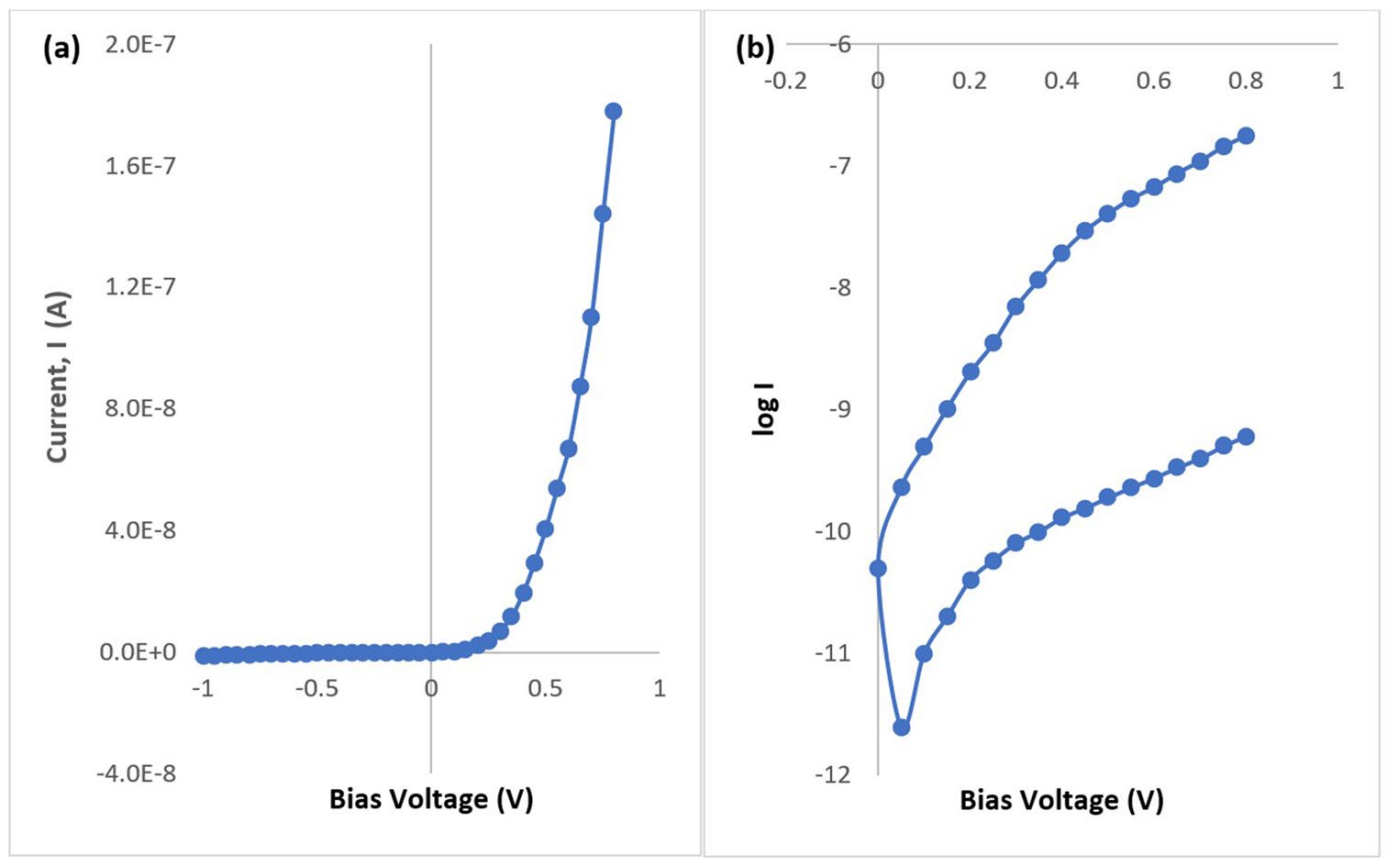
**(a)** Linear–linear I-V and **(b)** log-linear I-V characteristics of glass/FTO/p-CdMnTe/Al Schottky diodes under dark condition.

In order to further confirm the p-type electrical conduction of CdMnTe layers and to verify their electronic device quality and expected rectifying quality, glass/FTO/p-CdMnTe/Al structures were fabricated and examined. The layer that exhibited highest conductivity in section "DC conductivity measurement of CdMnTe layers" has been taken and 0.20 cm diameter circular Al contacts have been made using vacuum evaporation. As expected, p-CdMnTe/Al interface showed rectifying properties and typical dark I-V curves are shown in Fig. 10. With a series resistance (R_s_) 1.46 × 10^6^ Ω and shunt resistance (R_sh_) of 2.87 × 10^8^ Ω, these Schottky diodes depicted a rectification factor (RF = I_F_/I_R_) of ~ 10^2.5^ and an ideality factor (n) of ~ 1.19. The ideality factor indicates that the current transport mechanism is contributed by thermionic emission as well as recombination and generation (R&G)^33^. The measurements demonstrate that electroplated CdMnTe layers after CCT treatment forms rectifying Schottky barriers with Al and is suitable for electronic applications.

### Morphological study of CdMnTe layers

**Figure 11:**
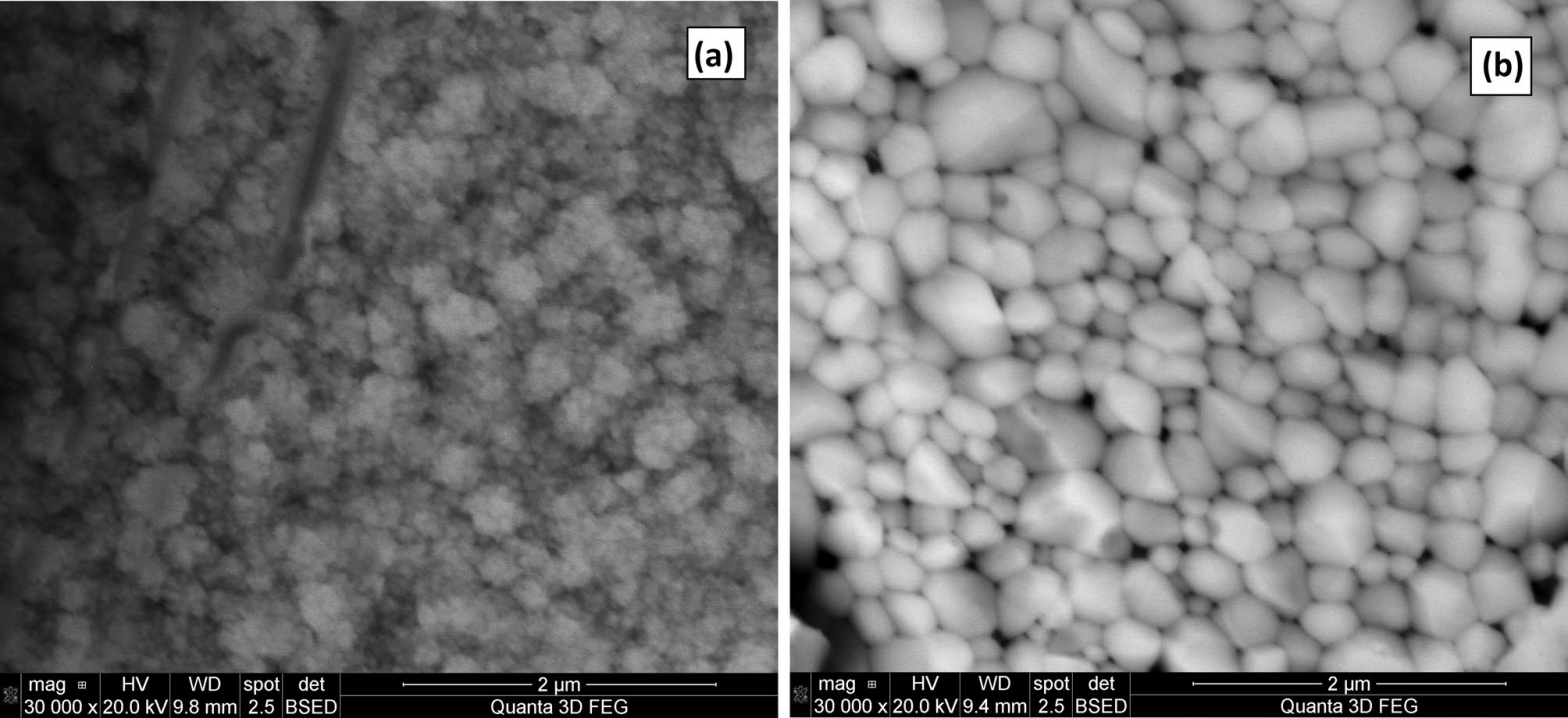
SEM micrographs of **(a)** AD-CdMnTe layers and **(b)** CCT-CdMnTe layers grown at 1430 mV cathodic potential.

In order to carry out the morphological characterisation of the CdMnTe layers, SEM analysis has been done in the form of micrographs with Quanta 3D FEG SEM instrument using 20.0 kV electron beam voltage and 30,000× of magnification. Figure 11 (a) is the SEM image of as-deposited CdMnTe layer. The grains exhibit small cauliflower-like nature when compared to CdCl_2_-heat-treated CdMnTe. Figure 11 (b) is the SEM image of CdCl_2_-annealed CdMnTe layer. Very large grains upto ≈0.80 μm were observed in the layers after the CCT treatment. This shows that agglomeration of crystallites turn into large grains following the CCT treatment.

## Discussion

When compared to the authors’ previous work on Mg incorporation in n-CdTe, Mn incorporation seems to have both added advantage and disadvantage. Both Mg and Mn converts n-CdTe layers into p-type layers as required. Bandgap widening also takes place with both Mg (up to ~ 2.80 eV) and Mn (up to ~ 2.20 eV) incorporation. However, Mg incorporation tended to reduce the crystallinity and form completely amorphous material layers. On the contrary, Mn kept the required poly-crystalline nature of the layers formed, hence posses to be more suitable for PV application.

Nevertheless, Photoluminescence (PL) study carried out on the ED CdTe:Mg layers previously showed that, Mg incorporation gives rise to intermediate defect levels though the optical absorption studies showed disappearance of typical CdTe absorption point (~ 1.45 eV) and widening of the material bandgap (~ 2.80 eV)^20^. On the other hand, electrodeposited CdMnTe layers clearly show mixed phases in the optical absorption study (section "Optical absorption study"), hence the possibility of the intermediate absorption point at ~ 1.45 eV acting as a defect state seems eminent.

The formation of ohmic contacts with high work function Au, and rectifying contacts with low work function Al, confirms the p-type electrical conduction of CdMnTe layers, and absence of Fermi level pinning at p-CdMnTe/Metal interface.

However, it should be noted that, unlike the other electrodeposited binary semiconductors, both CdTe:Mg and CdMnTe are electrodeposited ternary compounds and hence have a possibility to form mixed phased material which itself may come with additional pros and cons.

## Conclusion

The work carried out in this project focused on exploring electrodeposited CdMnTe thin films to use in novel graded bandgap multilayer solar cells based on p-type window materials. The work presented demonstrated for the first time that, Mn can be electrochemically incorporated into poly-crystalline CdTe successfully at a very low level. This Mn incorporation converts the n-CdTe layers into p-type CdMnTe layers with a wide bandgap of ~ 1.95 eV. Both DC conductivity and Schottky barrier studies carried out on the layers exhibit expected electrical properties and confirm their suitability to be applied in PV cell structures. However, the material shows mixed phased characteristic in optical absorption study, which is commonly exhibited in ternary compound previously electrodeposited for the same purpose.
